# Integrating Morphological and Physiological Responses of Tomato Plants to Light Quality to the Crop Level by 3D Modeling

**DOI:** 10.3389/fpls.2019.00839

**Published:** 2019-07-11

**Authors:** J. Anja Dieleman, Pieter H. B. De Visser, Esther Meinen, Janneke G. Grit, Tom A. Dueck

**Affiliations:** Business Unit Greenhouse Horticulture, Wageningen University & Research, Wageningen, Netherlands

**Keywords:** spectral composition of light, photomorphogenesis, functional-structural plant model, green light, blue light, red light

## Abstract

Next to its intensity, the spectral composition of light is one of the most important factors affecting plant growth and morphology. The introduction of light emitting diodes (LEDs) offers perspectives to design optimal light spectra for plant production systems. However, knowledge on the effects of light quality on physiological plant processes is still limited. The aim of this study is to determine the effects of six light qualities on growth and plant architecture of young tomato plants, and to upscale these effects to the crop level using a multispectral, functional-structural plant model. Young tomato plants were grown under 210 μmol m^-2^ s^-1^ blue, green, amber, red, white or red/blue (92%/8%) LED light with a low intensity of sunlight as background. Plants grown under blue light were shorter and developed smaller leaves which were obliquely oriented upward. Leaves grown under blue light contained the highest levels of light harvesting pigments, but when exposed to blue light only, they had the lowest rate of leaf photosynthesis. However, when exposed to white light these leaves had the highest rate of photosynthesis. Under green light, tomato plants were taller and leaves were nearly horizontally oriented, with a high specific leaf area. The open plant structure combined with a high light transmission and reflection at the leaf level allowed green light to penetrate deeper into the canopy. Plants grown under red, amber and white light were comparable with respect to height, leaf area and biomass production. The 3D model simulations indicated that the observed changes in plant architecture had a significant impact on light absorbance at the leaf and crop level. The combination of plant architecture and spectrum dependent photosynthesis was found to result in the highest rate of crop photosynthesis under red light in plants initially grown under green light. These results suggest that dynamic light spectra may offer perspectives to increase growth and production in high value production systems such as greenhouse horticulture and vertical farming.

## Introduction

Among the environmental factors in horticulture, light is one of the most important variables affecting plant development, growth and production (Kendrick and Kronenberg, 1994). In northern latitudes, light levels during winter periods are insufficient to maintain production levels and product quality, due to the low light intensities and short photoperiods (Davis and Burns, 2016). Therefore, natural light is supplemented by assimilation lamps, with high pressure sodium lamps (HPS) currently being the predominant greenhouse lighting source. However, the introduction of LED lighting systems has received considerable attention over the last decade. Their energy efficiency is higher compared to HPS, they can be positioned close to or within the canopy due to their low heat emission and they emit narrow-bandwidth light allowing the design and optimization of a dedicated light spectrum for plant growth and development (Morrow, 2008). However, before the full potential of LEDs as light source for plant production in greenhouses can be used, plant responses to spectral composition of the light have to be quantified.

The basic light color in most commercially used light sources in protected cultivation of plants is red light. Some blue light (ranging from 5 to 10%) is typically added to improve growth and prevent excessive stem elongation. Red light is considered to be the most efficient photosynthetically active light (McCree, 1972; Inada, 1976) as it is most readily absorbed by leaves and contains the least amount of energy per photon. Different areas of photosynthetically active radiation (PAR) between 400 and 700 nm and some wavelengths just outside of PAR, are perceived by plants through photoreceptors that promote specific developmental processes. This light-driven process is called photomorphogenesis and can alter plant morphology and architecture (Hernandez and Kubota, 2016), flower color and complex processes like flowering (Whitelam and Halliday, 2007). The commonly known influences of blue light on plant morphology are inhibition of leaf area and internode length resulting in a compact plant (cf. Hogewoning et al., 2010). Red light has been found to increase branching and stimulate bud outgrowth (Lötscher and Nösberger, 1997; Finlayson et al., 2010). Green light is thought to affect leaf morphology and orientation, so that the light can penetrate more deeply into the crop (Sun et al., 1998), and ultimately, increase photosynthesis and light utilization in lower leaf layers (Nishio, 2000; Kim et al., 2004; Folta and Maruhnich, 2007). Indeed, Kim et al. (2004) reported an increase in lettuce dry mass with increasing percentages of green light from zero to 24%. However, other studies reported no or inconclusive results on the effects of green light (Johkan et al., 2012; Hernandez and Kubota, 2016). When morphology and plant architecture are altered to increase light use efficiency in terms of higher light absorbance and utilization at the crop level, altering the light spectrum may enhance crop production (Huché-Thélier et al., 2016).

Most of the research performed to assess the effects of spectral composition on tomatoes has been performed with seedlings under LEDs as sole source lighting. In general, light color did not affect the development rate of tomato seedlings (expressed as the number of leaves) (Hogewoning et al., 2012; Wollaeger and Runkle, 2014; Snowden et al., 2016). Increasing the percentage of blue light from 10 to 28% reduced stem length, leaf area and dry matter, but did not affect the net assimilation rate (Snowden et al., 2016). When the green light percentage was reduced compared to white light (Went, 1957), tomato seedlings reached a higher dry mass. However, Snowden et al. (2016) did not find an effect of increasing the contribution of green light from 0 to 30% on leaf area or dry matter of tomato seedlings. They did exhibit shade characteristics such as an increased plant height and specific leaf area (Wang and Folta, 2013). Comparable effects were found when far red light was added to supplemental red and blue light under glasshouse conditions (Hogewoning et al., 2012). How these spectral effects translate to a full-grown, fruit producing crop, remains to be established.

An empirical establishment of the optimal spectral composition for a crop is nearly impossible due to the large number of trial combinations and the high investment of finances and time. Functional-structural plant models (FSPM) could be a more rapid and cost-effective solution in order to test the effect of various strategies varying in spectral composition of the light in the greenhouse (Vos et al., 2010; Henke and Buck-Sorlin, 2017). In order to do so, the model should simulate the light distribution of natural (diffuse and direct) daylight as well as artificial light within a realistic 3D representation of a crop (De Visser et al., 2014). So far, there are no functional-structural plant models available that have incorporated the spectral dependence of plant processes such as light absorption and photosynthesis. First steps were taken by Henke and Buck-Sorlin (2017) who have developed a full-spectrum ray tracer and incorporated that in the 3D modeling platform GroIMP. Application of this knowledge is crucial to understand and model the spectral effects on crop physiology, morphology and production.

In this paper, we aim to quantify the effects of a range of spectral light compositions on the development, architecture, photosynthesis and biomass accumulation of young tomato plants. While a red and blue light mixture is commonly used as supplemental LED lighting in greenhouse horticulture, separate red (663 nm) and blue (446 nm) lighting was chosen in this study to determine their photomorphogenetic properties. Furthermore, amber (627 nm) was added as an alternative wavelength to red light and green light (530 nm) because of the lack of knowledge on its effects in horticulture. A multispectral, functional-structural plant model (De Visser et al., 2014) was then used to integrate and upscale the impact of spectral differences of illumination to the level of the full-grown crop. Subsequently, the model was used to quantify the effects of the three main light colors (blue, green, and red) on light absorption and crop photosynthesis. The perspectives for a dynamic light recipe, using different spectra during crop growth and development in order to enhance yield of fruit vegetables are discussed.

## Materials and Methods

### Greenhouse Experiments

#### Plant Material and Climate Conditions

Tomato seedlings (*Solanum lycopersicum* cv. Ingar F1) were transplanted into well-watered rockwool blocks (10 × 10 cm) and supplied with a standard nutrient solution. Plantlets with 3–4 leaves were transferred into 2 air-conditioned greenhouses (24 m^2^), each with 3 ebb and flood tables (4.5 m^2^), one table for each of the 6 light treatments. Climate conditions were comparable in both greenhouses (Supplementary Table S1), with a 21/19°C day/night temperature regime at ca. 70% RH and 500 ppm CO_2_. The air temperature of each treatment was continuously measured with an internal NTC temperature sensor coupled to a Testo 174T data logger (Testo, Maharashtra, India). Plant temperature was measured using a hand-held thermo-hygrometer (Humiport 05 IR, E+E Electronic, Engerwitzdorf, Austria) on day 21 (experiment 1) and days 18, 23, and 30 (experiment 2). Measurements were taken hourly during the photoperiod on leaf 3 (counted from the top) of 12 plants per treatment. Air temperatures above the tables with white, red, blue, amber and red/blue LEDs were comparable at ca. 22°C, but was 1.0–1.7°C higher under green LEDs (Supplementary Table S2). The differences in leaf to air temperatures in all treatments remained similar, varying from 2.1 to 2.6°C, which means that not only the air temperature, but also the plant temperature was higher under green light. In a separate experiment, the air temperature under white and green LEDs was regulated in such a way that the plant temperatures were similar. A higher temperature was found to affect the number of leaves (0.5 more leaves in 3 weeks) and leaf length, but no effect on dry weight was found.

#### Light Treatments

The light treatments were performed twice for 21 days, in September–October 2014 and in December 2014–January 2015. Six spectral light treatments with an intensity of 210 μmol m^-2^ s^-1^ were provided with LEDs (Hortilux Schréder, Monster, Netherlands), with peaks at 446 nm (blue), 530 nm (green), 627 nm (amber), 663 nm (red), a red:blue mixture of 88:12 (commercial reference) and white as control (Supplementary Figure S1). White LEDs contained wave lengths between 400 and 700 nm in a ratio of 13% blue (400–500 nm), 55% green (500–600 nm) and 32% red (600–700 nm). Since the efficiency of green LEDs is much lower than that of the other colors, 28 green LED modules were used compared to 14 LED modules for the other colors in order to realize comparable light intensities in all treatments. In each repetition, the individual light treatments were randomly distributed over the 6 tables in the 2 greenhouse compartments. Light treatments were separated by opaque white plastic screens that did not transmit light between treatments. Of the 35 plants in each light treatment, 15 plants bordered the 20 replicates used for plant measurements. The developmental stage of the plants was recorded at the start of the treatments in order to separate treatment effects from those already present in the seedlings. Plants did not shade each other during the experiment; the plant density was 4.2 plants m^-2^. Prior to each experiment, the level of photosynthetically active radiation (PAR) was measured at plant height with a quantum sensor (LiCor 6400, Nebraska, NE, United States) at 36 spots per treatment to establish an even horizontal light distribution. The irradiance spectrum and intensity at 8 different positions in a horizontal plane in each treatment was measured with a Jaz single PAR-NIR spectrometer (Ocean Optics, Duiven, Netherlands). During these light measurements all screens were closed.

The LED modules were placed one meter above the plants and this height difference was maintained during the experiment. The LEDs were switched on 16 h before sunset and were turned off 1 h before sunset. During the experiment, the sun light intensity outside and just above the LED profiles was recorded every 5 min by PAR sensors. The artificial light intensity from the LEDs was set at 210 μmol m^-2^ s^-1^ during a 15 h day, a daily light sum of 11.3 mol m^-2^. The amount of sunlight was controlled with an OLS 60 screen (Gintec Shade Technologies, Vanessa, Ontario, Canada) on top of the greenhouse, an XLS SL 95 b/w Revolux screen (Ludvig Svensson, Kinna, Sweden) inside the greenhouse and blackout screens on the side walls. The maximum momentary sunlight intensity that was allowed, in addition to the light treatment intensities (210 μmol m^-2^ s^-1^) ranged between 4 and 75 μmol m^-2^ s^-1^. Averaged over the experiments, the contribution of solar radiation to the total light sum was 4.3% for experiment 1 and 2.3% for experiment 2.

#### Plant Measurements

To determine the effects of the different spectra, the rate of photosynthesis, stomatal conductance, leaf light reflection and transmission, concentrations of light capturing pigments, leaf orientation and total biomass production were measured.

##### Photosynthesis and stomatal conductance

During the last week of the experiments, rates of photosynthesis at 210 and 1500 μmol m^-2^ s^-1^ were determined on the 4th or 5th leaf (counted from the top) on 6 plants per treatment, using a light source providing 90% red and 10% blue light (LI-6400, LI-COR, Lincoln, Nebraska, NE, United States) as well as under 210 μmol m^-2^ s^-1^ ambient light (without the light source). The CO_2_ concentration in the cuvette was set at 500 ppm, the leaf temperature at 22°C and relative humidity at 60–75%. Stomatal conductance was determined together with the photosynthesis measurements.

##### Leaf light absorption

In the last week of each experiment, 8 single leaflets of the 4th or 5th leaf (counted from the top) per treatment were sampled, put in plastic bags with a wet tissue paper and transferred to a refrigerator for overnight storage. The next day, reflection (4 leaflets) and transmission (4 leaflets) of light in the range of 350–750 nm was measured in steps of 5 nm on the upper and lower side of the leaflets, using a spectrophotometer (Lambda 950 US/VIS, PerkinElmer, Waltham, MA, United States). Leaf light absorption was calculated as 1 – reflection – transmission. All values are presented as fraction of the incoming light.

##### Pigment analysis

At the end of each experiment, 3 leaf disks of 1 cm^2^ per plant were taken from the 5th or 6th leaf of 4 plants per treatment. The leaf disks were immediately frozen in liquid nitrogen and stored at -80°C until extraction. To release the pigments, leaf disks were transferred to glass vials with 3 mL N,N-dimethylformamide (Sigma-Aldrich, St. Louis, MO, United States). The vials were stored at -20°C and in darkness for at least 1 week prior to extraction. Then, absorbance at wavelengths 480, 646.8, 663.8, and 750 nm was measured using a spectrophotometer (Cary 4000, Agilent Technologies, Santa Clara, CA, United States). The concentrations of chlorophyll a, chlorophyll b and total carotenoids were calculated using equations from Wellburn (1994).

##### Crop architecture

During the last week of the experiments, leaf insertion angle (LIA), rachis (RA), top leaflet angle (TLA), base leaflet angle (base), middle leaflet angle (middle) and outside leaflet angle (outside) were measured on three leaves of six plants per treatment (Supplementary Figure S2). Leaflet elevation angles middle and outside were only measured in the second experiment. All leaf angles were measured by a protractor.

##### Plant biomass

At the end of each experiment, ten plants per treatment were harvested destructively. Each plant was split into plant organs already present at the start of the experiment and newly formed plant organs. A leaf larger than 2 cm in length was counted as a leaf. Internode length was measured between leaf 4 and 5. Leaf area was determined with a leaf area meter (LI-3100, LI-COR, Lincoln, Nebraska, NE, United States). Fresh and dry weights of all plant organs were determined. Dry weights were measured after drying leaves and stems for at least 48 h at 70°C. Dry matter content was calculated by dividing total dry weight by total fresh weight. The specific leaf area of leaf 6 was calculated by dividing its leaf area by its leaf dry mass.

### 3D Model Simulations

#### Model Set-Up and Implementation of Spectral Dependencies

The 3D model constructed within GroIMP (Growth Grammar-related Interactive Modeling Platform) (Kniemeyer, 2008) was used for the simulations. This functional structural model entailed a virtual reconstruction of a 3D static tomato crop, the enclosing greenhouse structure and the LED fixtures. The virtual greenhouse was based on an existing greenhouse using the positions, shapes and optical properties of all the objects (roof, walls, lamps, gutter, plants) in a realistic 3D scene as described by De Visser et al. (2014). Light distribution was computed by the GroIMP radiation model, which is based on an inversed Monte Carlo path tracer, similar to that used by Cieslak et al. (2008). The spectral distribution of the light can be modeled in 5 nm intervals in the photosynthetic active radiation (PAR) range (400–700 nm) with a simultaneous simulation of all intervals. Net photosynthesis was simulated for each leaflet based on absorbed light, air temperature and CO_2_ according to Kim and Lieth (2003), with a leaf age dependent value for maximum rate of electron transport (J_max_ in μmol electrons m^-2^ s^-1^).

The effects of spectral light composition were incorporated in the model via (1) light use efficiency of leaf photosynthesis, (2) optical properties of the leaves, and (3) plant architecture. (1) Differences in light response depending on the spectral composition were quantified following McCree (1972), and using previously obtained data of the light use efficiency for 9 colors in the PAR range (Snel et al., 2011). Subsequently, the value of α in the photosynthesis model was tuned to the observations, thus quantifying the number of micromoles assimilated CO_2_ per micromole absorbed light, i.e., the light use efficiency at leaf level. (2) The optical properties of the leaves entailed their measured light reflection and transmission and the calculated light absorption of PAR for the light colors used (blue, green, and red). (3) The model was parameterized based on plant architecture measurements (internode length, leaf length, leaf elevation angles and leaf curvature) in the greenhouse experiments. Parameters were assumed valid only for similarly aged young leaves, while properties of the other age classes of leaves were derived from these parameter values through an age-dependent function as determined in De Visser et al. (2014).

#### Upscaling to Crop

The hemispherical emission pattern of top light placed above plant rows at 10° intervals matched well with that of an existing 250 cm commercially available LED lamp (Philips GreenPower) with light emission calibrated to 100 μmol m^-2^ s^-1^ per color. The LED modules were positioned 4.75 m above the ground above each double row of plants in line with the rows. The mathematical approach for the calculation of the 3D emission pattern is described in Buck-Sorlin et al. (2009). The crop was represented as a static structure, following measurements on tomato cv. Komeett (De Visser et al., 2014). Each plant in this crop consisted of 8 trusses and 21 composite leaves, each leaf being composed of 15 leaflets of a fixed geometry. The modeled scene on 3 × 3.2 m ground area consisted of 32 plants. Plants were placed on slab pairs 0.8 m above the floor, 0.4 m apart and divided by a path, with a total of 1.6 m distance from center to center between slab pairs. The tops of the plants were situated 3.5 m above the floor. An infinite canopy was simulated by placing perfect mirrors around the scene.

The model scenarios entailed (1) the spectral composition of the light, and (2) the plant architecture. For each scenario, the total light absorption by the crop and crop photosynthesis was calculated by aggregating the light absorption and photosynthesis of each individual leaflet in the crop. The spatial light distribution, reflection and absorption by the tomato crop was simulated for blue (400–480 nm), green (500–550 nm) and red (600–700 nm) light according to spectral output of the LEDs modules used in the experiment. The simulated output level of the LEDs was 100 μmol m^-2^ s^-1^ for each color separately. Simulations were performed for plants with one, seven or twenty-one leaves, illustrating the effect of upscaling from leaf to young plant to a full crop with leaf area index (LAI) of 3. Architectural changes resulting from the light treatments were implemented in the 3D model.

Plant morphological changes resulting from the light treatments were (1) simulated at leaf scale by positioning one leaf with the changed morphology (leaf rachis angles, angles of leaflets within the leaf) directly under, and parallel to, a string of white LED lights; and (2) extrapolated to crop level by simulation of light interception and photosynthesis of a full-grown crop. For (1), the effect of the morphological changes of all 6 light treatments on light interception was simulated. For (2), in total eighteen scenarios were run, comprising of scenario runs of illumination with blue, green and red light (3x) at 3 hierarchical scales (leaf, young plant, crop), and a full grown crop grown under blue, green or red light with changes in plant architecture and quantum efficiency induced by those light colors (3x) transferred to blue, green and red light. Each scenario was carried out using 20 million light rays in the ray tracer and 40 recursions (light bounces).

### Statistical Analysis

The experiments were repeated sequentially and the data are presented as means per treatment. The experimental design was a randomized block design with time as block and different light compositions as treatments. Fisher’s unprotected least significance test was used to make *post hoc* multiple comparisons among means from significant analysis of variance (ANOVA) tests. *P*-values smaller than 0.05 were considered as significant different for the pairwise comparisons. For the statistical analysis GenStat (17^th^ edition) was used.

## Results

### Greenhouse Experiments

#### Plant Morphology and Biomass Accumulation

**FIGURE 1:**
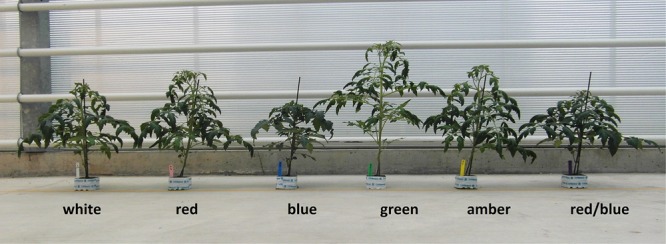
Effect of spectral composition of the light on plant morphology of tomato plants grown under white, red, blue, green, amber and red/blue LED light during 21 days.

**FIGURE 2:**
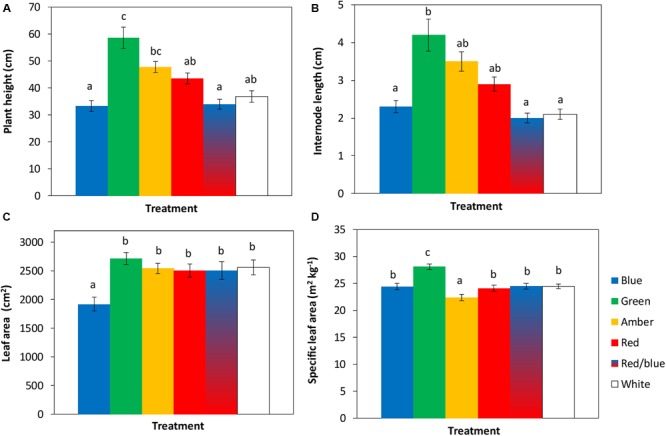
Effects of spectral composition of the light during 21 days on plant height **(A)**, internode length **(B)**, leaf area **(C)** and specific leaf area (SLA) of the 6th leaf **(D)**, recorded at the end of the experiments (*n* = 2, average of 10 plants). Vertical bars indicate the standard error of mean (*n* = 2). Different letters indicate significant differences (*P* < 0.05).

**FIGURE 3:**
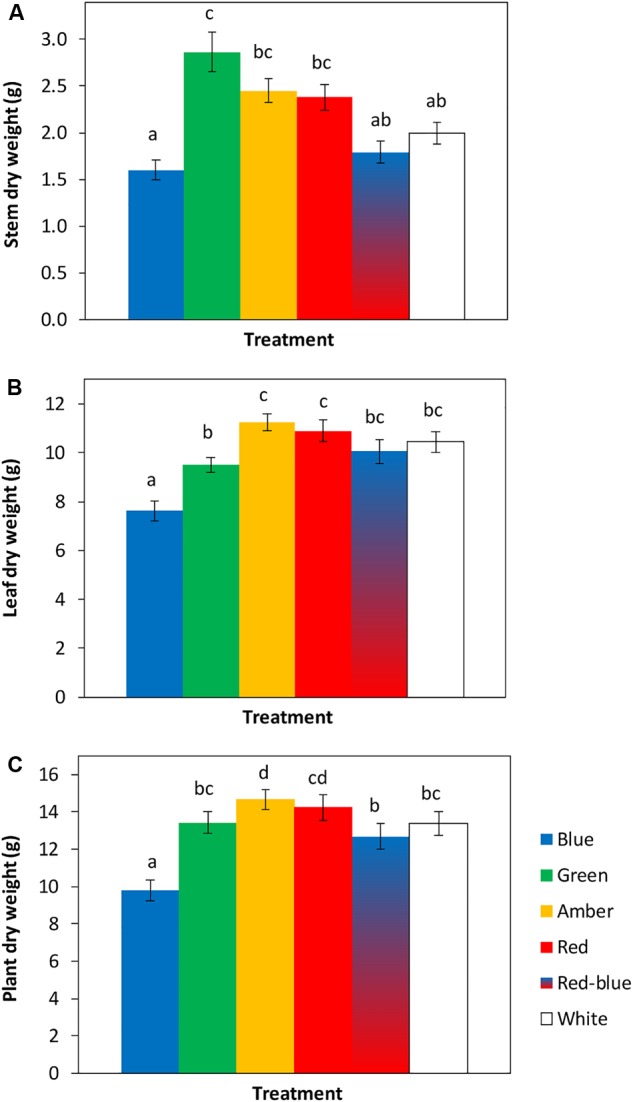
Effects of spectral composition of the light during 21 days on dry weights of the stem **(A)**, leaves **(B)** and total plant (**C**, excluding roots) (*n* = 2, average of 10 plants). Vertical bars indicate the standard error of mean (*n* = 2). Different letters indicate significant differences (*P* < 0.05).

The tomato seedlings had 3 to 4 leaves when light treatments started, a height of 9 or 14 cm and plant dry weight of 0.07 or 0.19 g, respectively, in experiments 1 and 2. After 3 weeks of treatments, effects on plant morphology were visible (Figure 1). Plants grown under green light were significantly taller than plants grown under blue, red, red/blue and white light, due to their higher average internode length (Figure 2). Leaf area of plants grown under blue light was significantly lower than under the other colors. SLA of plants grown under green light was highest, and under amber light significantly lower than in the other treatments (Figure 2). Total plant biomass after 3 weeks of cultivation was the highest for plants grown under amber and red light, whereas plants grown under blue light had the lowest leaf dry weight and total plant dry weight (Figure 3).

To quantify the observed changes in morphology, leaf and leaflet angles of 3 newly formed leaves in each light treatment were measured. Leaf lengths and widths were largest for plants grown under green, amber and red light, followed by white, red/blue and blue light (Table 1). Leaf insertion angle and rachis angles were highest for plants grown under blue light, resulting in a more upright position. Leaves grown under green light had a rachis angle close to 0°, a nearly horizontal orientation, whereas the rachis angles of leaves grown under amber and red light were negative. Top leaflet angles were most negative for blue, and least for plants grown under green light (Table 1). Outside leaflets developed under green, amber and red light were bent more downward compared to leaves grown under blue, red/blue and white light (Table 1).

**Table 1:** Effects of spectral composition on leaf architecture.

Treatment	Length (cm)	Width (cm)	LIA (°)	RA (°)	TLA (°)	Base (°)	Middle (°)	Outside (°)
Blue	30 a	29 a	67 a	14 d	–41 a	15 b	9	–39
Green	38 d	40 cd	82 bc	–3 bc	–24 c	14 b	4	–57
Amber	38 d	41 d	87 c	–18 a	–31 bc	7 a	4	–56
Red	37 cd	40 d	84 bc	–12 ab	–30 bc	7 a	5	–52
Red/blue	35 b	35 b	74 ab	8 cd	–34 ab	10 ab	5	–44
White	36 bc	38 c	75 abc	9 cd	–30 bc	12 ab	3	–43

*Spectral compositions are blue, green, amber, red, red/blue (95/5%) and white light, maintained during 21 days on the mean leaf length, leaf width, leaf insertion angle (LIA), rachis angle (RA), top leaflet angle (TLA) and base leaflet angle (base) (*n* = 2, measured on 3 leaves of 6 plants), the mean middle leaflet angle (middle) and outside leaflet angle (outside) (*n* = 1, measured on 3 leaves of 6 plants in experiment 2). Different letters within columns indicate significant differences (*P* < 0.05).*

#### Light Absorption and Photosynthesis

**FIGURE 4:**
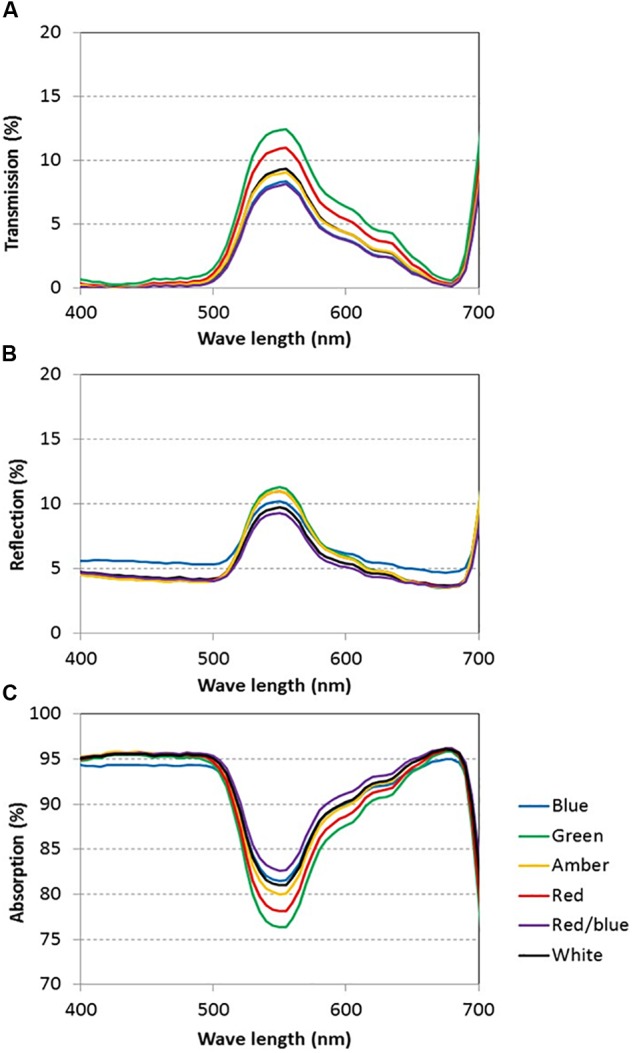
Light transmission **(A)**, reflection **(B)** and absorption **(C)** of tomato leaves grown under blue, green, amber, red, white, and red/blue light during 21 days (*n* = 2, average of 4 leaves).

Light absorption values were derived from reflection and transmission measurements between 400 and 700 nm of the adaxial side of the tomato leaves (Figure 4). Reflection and transmission values peaked around 550 nm, reducing the light absorption of all light colors in that range. Plants grown under green light had the highest transmission in all wavelengths, whereas plants grown under blue and red/blue light had the lowest transmission (Figure 4A). Reflection was highest for leaves grown under blue light, except for wavelengths around 550 nm (Figure 4B). The absorption of blue light was lowest for plants grown under blue light (Figure 4C) and the absorption of green light was lowest for plants grown under green light. The fraction of PAR absorbed by a tomato leaf under different light colors was then calculated by multiplying the absorption characteristics of the leaf from Figure 4 with the spectrum of that treatment (Supplementary Figure S1) in the range of 400 to 700 nm. The highest fraction of light was absorbed by leaves grown under red, blue and red/blue light, while leaves grown under green light absorbed the lowest fraction of light (Table 2). The concentration of chlorophyll a and b and carotenoids in the leaves grown under blue and red/blue light was higher (Table 3) than those under green, amber and red light. The ratio chlorophyll a:b was significantly higher for plants grown under blue LEDs.

**Table 2:** Effects of spectral composition on the fractions of light absorbed.

Treatment	Fraction light color absorbed	Fraction white light absorbed
Blue	0.94	0.89
Green	0.82	0.87
Amber	0.91	0.89
Red	0.94	0.88
Red/blue	0.95	0.90
White	0.89	0.89

*Fraction of light of the treatment color absorbed is calculated by multiplication of absorption characteristics of the leaves by the spectrum of that treatment. Fraction white light absorbed is calculated by multiplication of absorption characteristics of the leaves by the spectrum of white light.*

**Table 3:** Effects of spectral composition on the light on chlorophyll contents.

Treatment	Chlorophyll a (g m^-2^)	Chlorophyll b (g m^-2^)	Ratio of chlorophyll a:b	Carotenoids (g m^-2^)
Blue	0.52 b	0.13 b	4.00 b	0.11 c
Green	0.39 a	0.10 a	3.83 a	0.08 a
Amber	0.42 a	0.11 ab	3.73 a	0.09 ab
Red	0.40 a	0.10 a	3.85 a	0.08 a
Red–Blue	0.51 b	0.13 b	3.87 ab	0.10 c
White	0.47 ab	0.13 ab	3.75 a	0.09 bc

*Spectral compositions are blue, green, amber, red, red/blue (95/5%) and white light, maintained during 21 days on contents of chlorophyll a, chlorophyll b, chlorophyll a:b ratio and carotenoids (*n* = 2, measured on 3 leaf disks of 4 plants). Different letters within columns indicate significant differences (*P* < 0.05).*

To determine the *in situ* rate of photosynthesis and the effect of leaf characteristics on the rate of photosynthesis, measurements were taken at ambient conditions and under the red/blue light of the LI-6400 portable photosynthesis equipment. Stomatal conductance was higher for plants grown under blue LEDs compared to the other light colors (Table 4), both when measured under ambient light conditions and under red/blue light. The rate of photosynthesis under ambient light was lowest for plants grown and measured under blue light, followed by that of leaves under green light and red light (Table 4). When the combination of red/blue LEDs in the light source of the LI-6400 was used, the photosynthetic rate of plants grown under blue light was significantly higher than of the other light colors, followed by plants grown under red/blue and white light (Table 4).

**Table 4:** Effects of spectral composition of the light on rate of photosynthesis and stomatal conductance.

	Measured under ambient light conditions		Measured under red/blue light of the LI-6400	
Treatment	Rate of photosynthesis (μmol CO2 m^-2^ s^-1^)	Stomatal conductance (mol H_2_0 m^-2^ s^-1^)	Rate of photosynthesis (μmol CO2 m^-2^ s^-1^)	Stomatal conductance (mol H_2_0 m^-2^ s^-1^)
Blue	6.2 a	0.72 b	11.6 c	0.72 b
Green	7.0 b	0.45 a	10.3 a	0.44 a
Amber	8.3 d	0.49 a	10.3 a	0.42 a
Red	7.6 c	0.42 a	10.4 a	0.38 a
Red–Blue	8.2 d	0.54 a	10.9 b	0.50 a
White	8.2 d	0.50 a	10.9 b	0.38 a

*Spectral compositions are blue, green, amber, red, red/blue (95/5%) and white light, maintained during 21 days on rates of photosynthesis and stomatal conductance measured under ambient light conditions or under the standard red/blue light (90/10%) in the cuvette of the LI-6400 (*n* = 2, average of 6 plants). Measuring conditions in the cuvette were 500 ppm CO_*2*_, leaf temperature of 22°C and relative humidity of 60–75%. Different letters within columns indicate significant differences (*P* < 0.05).*

### Integration by Means of 3D Model Simulations

**FIGURE 5:**
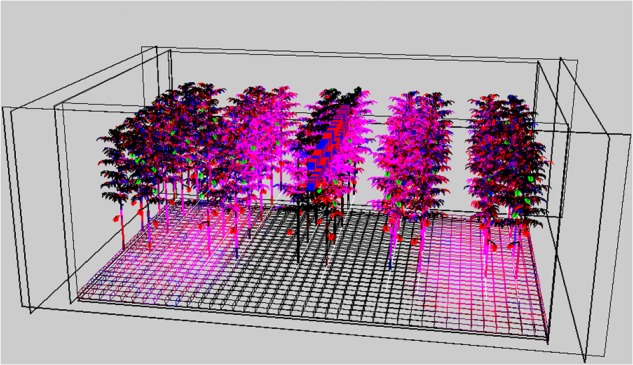
Simulated image of the 3D crop where the intensity of the pink color illustrates the light distribution of 95% red and 5% blue LEDs placed directly above the middle row.

The plant architecture data obtained in the experiments were used to parameterize the 3D model. Absorbance of white light by a leaf grown under the various spectra was modeled relative to the absorbance of a leaf grown under white light (Table 2). The more upward position of leaves and associated leaflets of plants grown under blue light decreased light capture, whereas the nearly horizontal position of the leaf (rachis angle close to 0°) of leaves grown under green light increased the interception of light (Table 2). The visualization of the results in Figure 5 shows that not all of the light was intercepted by the crop. However, light reaching the greenhouse floor was less than 10% of the input since also canopy reflection and possible losses to greenhouse objects (wires, slabs, etc.) occurred.

#### Scenarios of Illumination With Various Spectra on Light Interception and Photosynthesis

In the first set of scenario runs, effects of illumination with blue, green and red light on light absorbance and instantaneous rate of photosynthesis (3x) at 3 hierarchical scales (leaf, young plant, crop) were calculated. The absorbance of green light at leaf level was found to be lower than that of red and blue light (Table 5). The relative differences in light absorbance between blue, green and red light decreased with increasing number of leaf layers. The simulated absorbance of light indicates that 2% more blue and red light than green light is absorbed at the crop level. The photosynthetic response of a crop to a given amount of light is the result of both absorbance and quantum efficiency. Red light had the highest rate of gross photosynthesis (Table 5), due to the higher quantum efficiency (Table 4) and a high light absorption. Blue light resulted in a lower rate of photosynthesis than red or green light, since wavelengths above 500 nm (green, amber, and red) allow for a higher quantum efficiency and thus higher crop photosynthesis.

**Table 5:** Modeled light absorbance, gross photosynthesis and light use efficiency.

	Plant/crop grown under white		
	light and exposed to:		
	Blue light	Green light	Red light
**Light absorbance (% of input)**
Leaf level	14.1	13.3	13.8
Plant level	58.2	55.3	57.4
Crop level	90.1	88.3	89.9
**Gross photosynthesis (μmol CO_2_ m^-2^ floor s^-1^)**			
Leaf level	0.742	0.756	0.845
Plant level	3.44	3.59	4.01
Crop level	5.28	5.62	6.19
**Light use efficiency (μmol CO_2_ mmol^-1^ absorbed light)**			
Leaf level	52.7	57.0	61.1
Plant level	59.1	64.9	69.9
Crop level	58.6	63.7	68.8

*Model calculations for plants grown under white light and then exposed to 100 μmol m^-*2*^ floor s^-*1*^ incident blue, green or red light, for the leaf, plant and crop level. Light use efficiency is calculated as the gross photosynthesis in μmol CO_*2*_ m^-*2*^ floor s^-*1*^ divided by the amount of absorbed light (mmol m^-*2*^ floor s^-*1*^).*

#### Light Interception and Photosynthesis at the Crop Level, for a Crop Grown Under Blue, Green or Red Light

In the second set of scenario runs, simulations were performed with a virtual crop grown under blue, green or red light. With the induced changes in plant architecture and quantum efficiency of photosynthesis, crop light interception and photosynthesis was determined. The simulated experiment lasted 10 weeks and started at planting with 4 leaves per plant. The absorbance of green light was lower than the absorbance of red or blue light, independent of the light color under which the plants were grown (Table 6), and was similar to the results of the crop grown under white light (Table 5). The effect of morphology on light absorbance at the crop level was large. Plants that developed under green light were modeled to be taller than plants grown under blue and red light and had a larger leaf area, resulting in the highest light absorption for green-grown plants, even under the somewhat more reflected green light. Virtual plants grown under blue light, contrary to green, were very compact with smaller leaves, shorter internodes and leaves positioned upward, intercepting approximately 25% less light relative to red or green light grown plants (Table 6).

**Table 6:** Modeled light absorbance, gross photosynthesis and light use efficiency of a tomato crop.

	Plants exposed to:		
	Blue light	Green light	Red light
**Light absorbance (% of input)**
Plants grown under blue light	77.3	74.3	76.1
Plants grown under green light	93.7	91.5	93.2
Plants grown under red light	90.2	87.7	89.4
**Gross photosynthesis (μmol CO_2_ m^-2^ floor s^-1^)**			
Plants grown under blue light	3.88	4.12	4.59
Plants grown under green light	5.90	6.18	6.92
Plants grown under red light	4.79	5.04	5.64
**Light use efficiency (μmol CO_2_ mmol^-1^ absorbed light)**			
Plants grown under blue light	50.2	55.5	60.3
Plants grown under green light	63.0	67.5	74.3
Plants grown under red light	53.1	57.5	63.1

*Model calculations for a tomato crop grown under blue, green or red light and then exposed to 100 μmol m^-*2*^ floor s^-*1*^ incident blue, green or red light. Light use efficiency is calculated as the gross photosynthesis in μmol CO_*2*_ m^-*2*^ floor s^-*1*^ divided by the amount of absorbed light (mmol m^-*2*^ floor s^-*1*^).*

The light absorbed was converted into assimilates using the FSPM model following the observed action spectrum. This action spectrum resulted in a higher photosynthesis and assimilate production per photon absorbed red light, which increased crop photosynthesis for the scenarios with red light, relative to that of blue and green light (Table 6). The differences in absorbance between green and blue grown plants were almost fully reversed by spectrally dependent photosynthesis, resulting in a better utilization of the green light relative to blue. Both absorbance and crop photosynthesis of red light were higher than for green light and crop photosynthesis was higher than for blue light (Table 6). The combination of plant architecture and spectrum dependent photosynthesis was found to result in the highest light use efficiency of green light grown plants that received red light.

## Discussion

In this study, the effects of a range of spectral compositions of LED light on growth and development of young tomato plants were quantified. These results were up scaled to the crop level, using a multispectral, functional-structural plant model. These 3D model calculations indicated that a combination of plant architecture and spectrum-dependent photosynthesis favored crop photosynthesis of plants grown under green light compared to plants grown under blue or red light. Crop photosynthesis was highest under red light in plants initially grown under green light. The observations underlying these calculations and the implications of these results are discussed below.

### Spectral Quality Affects Crop Morphology and Light Absorption

Spectral composition of the light was found to alter crop morphology of young tomato plants, in terms of stem and internode lengths, leaf area and leaf angles. Compared to the white light treatment, plants grown under green light were significantly taller, supporting observations from Folta and Maruhnich (2007). They found that even low intensities of green light stimulate early stem elongation, possibly by opposing or inhibiting the blue light influence on stem elongation (Wang et al., 2015). The specific leaf area (leaf area per g leaf dry weight) of plants grown under green light was larger than under the other light colors which is in accordance with earlier observations (Casal et al., 1987; Wang et al., 2015). Leaf lengths and widths under green, amber and red light were larger than under white and blue light, in agreement with results of Wollaeger and Runkle (2014). The combination of shoot elongation, high specific leaf area and large leaves under green light resembles a phenotype with a shade avoidance response (Ballaré and Pierik, 2017). Consequently, a taller, more open plant structure developed under green light allows for more light penetration into the lower canopy layers and thus a higher canopy light interception (Sarlikioti et al., 2011). The effects of blue light on plant morphology opposed those of green light: plants grown under blue light were shorter and had smaller leaves. Leaf area of plants grown under blue light was thereby significantly lower than that of plants grown under the other light colors, which confirms earlier results of Snowden et al. (2016) and Hernandez and Kubota (2016). Leaf orientation of plants grown under blue, red/blue and white light was more upright than under green, red or amber. Inoue et al. (2008) also found that even under weak blue light newly formed leaves were obliquely formed upward. When Arabidopsis plants were given 100 μmol/m^2^/s blue, green or red light, Mullen et al. (2006) observed the highest leaf inclination for blue, followed by green and red light, which agrees with our observations.

Leaf light absorption is mediated by photosynthetic pigments which capture light to drive photosynthesis. Since chlorophyll a and b are the primary photosynthetic pigments with maxima under blue and red colors (Atwell et al., 1999), and carotenoids harvest mainly blue light (cf. Nisar et al., 2015), leaf light absorbance was expected to differ between treatments. The chlorophyll and carotenoid contents were expected to be enhanced, especially under blue light (Johkan et al., 2010; Wang et al., 2014) as their production is stimulated by cryptochromes (Weller et al., 2001). The content of these photosynthetic pigments under blue, red/blue and white was indeed higher, due to the fact that these 3 colors contain varying degrees of blue in their spectrum. This confirms observations by Hogewoning et al. (2010), who found that the chlorophyll content was enhanced by blue rather than red light. Red and blue photons are more readily absorbed than other colors (Possart et al., 2014; Wang et al., 2015; Fraser et al., 2016), supporting observations in this study that leaves absorbed 94% of the red and blue light compared to the 82% of the green light. The large difference in absorbance of red and blue versus green light is partly due to the higher transmission of green light through the leaf, supporting earlier observations (Kim et al., 2004; Bugbee, 2016; Fraser et al., 2016). Green light penetrates deeper into the canopy which is beneficial for light absorption at lower leaf levels (Sun et al., 1998; Smith et al., 2017) and thereby for crop light absorption. The observed effects of light colors on light absorbance and plant morphology were incorporated in the 3D model and upscaled to crop level. The results of the model scenarios confirmed that differences in absorption of green and blue light reduced when moving from leaf to crop level. At crop level, the altered plant architecture were calculated to result in a higher light absorbance by a crop grown under green light (93%) than under red light (90%) and blue light (77%).

### Spectral Quality Affects Crop Photosynthesis and Biomass Accumulation

Once absorbed, light will be utilized for photosynthesis. Photosynthesis measurements were performed under ambient levels of each light color and under the artificial LI-6400 light source (red/blue LEDs). Despite the enhanced chlorophyll and carotenoid content in plants grown under blue light, the photosynthetic rate of plants grown and measured under blue light was lowest, followed by that of leaves grown and measured under green light and then by plants grown and measured under red light, which agrees with the response curves of McCree (1972) and Inada (1976). Up-scaling these results to the crop level with the 3D model also showed an increased rate of gross photosynthesis for plants exposed to red light compared to green and to blue light. However, when measured under a standard light spectrum, the photosynthetic rate of plants grown under blue light was found to be significantly higher than that of the other light colors. Apparently, the increased concentration of light absorbing pigments in leaves has considerable consequences for leaf CO_2_ uptake under light conditions that deviate from pure blue.

Plant dry weights under red and amber light were higher than in the other treatments, which is agreement with previous results of Wollaeger and Runkle (2014) who also found a higher plant dry weight under 100% red light compared to blue light. Plants grown under green light had similar shoot dry weights to those grown under red and white light, in spite of their lower light absorption at leaf level. This was apparently compensated for at the crop level, where lower leaf layers absorbed relatively more green light (Sun et al., 1998; Nishio, 2000), resulting in comparable shoot dry weights (Smith et al., 2017). Plant dry weight was significantly lowest in the treatment with blue light, most likely due to their smaller leaf area and shorter stems resulting in a compact plant and corroborating earlier findings (cf. Whitelam and Halliday, 2007; Huché-Thélier et al., 2016). The lower rate of instantaneous photosynthesis at the leaf level under blue light also observed by Hogewoning et al. (2010) appears to be compensated for by other factors like thicker leaves, an enhanced chlorophyll content and reduced leaf area, resulting in less photosynthesis at the crop level. It appears that blue light stimulates the production of photosynthetic pigments, but that they are better utilized under other light colors. The reduced leaf area resulted in a reduction in light interception, less crop photosynthesis and thereby in a significantly lower total plant biomass. This is in agreement with Bugbee (2016) who concluded that the fraction of light intercepted by a crop is more closely related to biomass than the short-term effects on quantum efficiency of photosynthesis. The plants in this experiment were exposed to blue light during the day, but the question arises how long the leaves should be exposed to blue light to enhance pigment content and rate of photosynthesis without compromising leaf area. This may well offer some perspective for increasing the production of greenhouse crops by applying a dynamic light recipe during the day.

### Using Different Light Colors Can Increase Crop Production

The experimental results of this study were incorporated into the 3D model and up-scaled to crop level to quantify the effects of different light colors on crop yield and production. The model simulations indicated that the observed changes in plant architecture had a significant impact on light absorption at the leaf and crop level, with light absorbance being the highest for plants grown under green and red light compared to blue light. The differences in light absorbance decrease with increasing leaf area index, i.e., from leaf to crop level. For a single leaf, the reflection for green is ca. 10% higher than that of red light. However, this has a relatively small impact on light absorption at the crop level. This implies that red and blue light are better utilized in the upper leaf layers, while green light penetrates more deeply into the canopy before being absorbed, as stated earlier by Paradiso et al. (2011). The 3D model simulated that approximately 2% more blue than green light is absorbed at the crop level. However, this is compensated for by the larger crop leaf area under green light compared to that under blue light. Thus, in order to increase the leaf area and thereby the light use efficiency at crop level, green light could be added to the currently used LED red/blue spectrum. In fruit bearing tomato plants, increasing the fraction of green light to 41% was found to increase total plant biomass and fruit yield compared to red/blue LEDs in the presence of sunlight (Kaiser et al., 2019).

Following the observed action spectrum, the 3D model predicted a higher rate of photosynthesis per photon absorbed red light, compared to blue and green light. Interestingly, the differences in absorbance between green and blue grown plants were almost fully reversed by spectrally dependent photosynthesis, showing a better utilization of the green light at the crop level despite its lower absorbance at the leaf level relative to blue. Both absorbance and assimilation of the red light is better than that of green and blue. The combination of plant architecture and spectrum dependent photosynthesis was found to result in the highest light use efficiency of plants grown under green light that received red light. These results suggest that differences in morphological and physiological characteristics in tomato grown under different spectra can have important consequences for tomato crop growth and production. The plants in our experiments were grown for 3 weeks under a continuous light spectrum, resulting in alterations in crop morphology and thereby light absorption. However, short term changes in spectrum will affect instantaneous rate of photosynthesis. Thereby, our results suggest that dynamic light spectra that vary during the day offers perspectives to increase growth and production in high value production systems, such as greenhouse horticulture and vertical farming.

In this study, we used a functional structural plant model to upscale experimental results at the leaf and young plant level to the level of a fruit-bearing crop level. These 3D models can be valuable tools to predict the crop’s response to a dynamic light recipe, since they have shown to reproduce architectural responses to spectral stimuli like changed red:far-red ratio well (Evers et al., 2007; Kahlen and Stützel, 2011, Bongers et al., 2018). In our study, the response to dynamic spectral lighting could not be predicted due to the static nature of the spectral treatments. Therefore, time constants of processes or time-dependent dose-response relationships could not be parametrized. The significant effects of light colors on plant architecture justify the experimental quantification of the time constant of these responses. Such an improved model functionality will help to run scenarios to get a better idea of the crop’s response to different dynamic light recipes.

## Author Contributions

JD, EM, and TD conceived and designed the experiments. EM and JG executed the experiments and analyzed the data. PV adapted the 3D model and did the scenario study. JD, PV, and TD wrote the final version of the manuscript.

## Conflict of Interest Statement

The authors declare that the research was conducted in the absence of any commercial or financial relationships that could be construed as a potential conflict of interest.
