# Supplementary material for: Integrating Morphological and Physiological Responses of Tomato Plants to Light Quality to the Crop Level by 3D Modeling
Source: Front Plant Sci. 2019 Jul 11;10:839. doi: 10.3389/fpls.2019.00839 (PMC6637845; doi:10.3389/fpls.2019.00839)
Supplement: Supplementary file 1 [file Table_1.pdf]

Supplementary Material

Integrating Morphological and Physiological Responses of Tomato Plants to Light Quality to the Crop Level by 3D Modelling

J.A. Dieleman*, P.H.B. De Visser, E. Meinen, J.G. Grit, T.A. Dueck

*** Correspondence:** J.Anja Dieleman, anja.dieleman@wur.nl

Table S1 | Greenhouse climate.

	**Air temperature (°C)**	**CO_2 concentration (ppm)**	**Vapour deficit (g m^{-2})**
Experiment 1			
Greenhouse 1	20.3	509	5.12
Greenhouse 2	20.4	506	5.06
Experiment 2			
Greenhouse 1	20.1	539	5.49
Greenhouse 2	20.9	524	6.19

Realized air temperatures, CO_2 concentrations and vapor deficits in the 2 greenhouse compartments in the 2 experiments. Data are the means of 5 min recordings during the experiments (n=6048).
