# Supplementary material for: Integrating Morphological and Physiological Responses of Tomato Plants to Light Quality to the Crop Level by 3D Modeling
Source: Front Plant Sci. 2019 Jul 11;10:839. doi: 10.3389/fpls.2019.00839 (PMC6637845; doi:10.3389/fpls.2019.00839)
Supplement: Supplementary file 2 [file Table_2.pdf]

Supplementary Material

Integrating Morphological and Physiological Responses of Tomato Plants to Light Quality to the Crop Level by 3D Modelling

J.A. Dieleman*, P.H.B. De Visser, E. Meinen, J.G. Grit, T.A. Dueck

*** Correspondence:** J.Anja Dieleman, anja.dieleman@wur.nl

TABLE S2 | Effects of spectral composition on air and leaf temperatures.

Treatment	Air temperature (°C)	Leaf temperature (°C)	Temperature difference (°C)
Blue	22.1 ± 0.1	19.6 ± 0.2	2.5 ± 0.3
Green	23.4 ± 0.1	20.9 ± 0.2	2.6 ± 0.2
Amber	22.4 ± 0.1	19.9 ± 0.2	2.5 ± 0.2
Red	21.7 ± 0.1	19.5 ± 0.2	2.2 ± 0.2
Red/blue	21.6 ± 0.1	19.4 ± 0.2	2.1 ± 0.2
White	22.0 ± 0.1	19.6 ± 0.1	2.3 ± 0.2

Realized air temperatures, leaf temperature and the difference between leaf and air temperatures in each of the spectral light treatments measured on 4 days in divided over experiments 1 and 2 (n=22).
