# Supplementary material for: Integrating Morphological and Physiological Responses of Tomato Plants to Light Quality to the Crop Level by 3D Modeling
Source: Front Plant Sci. 2019 Jul 11;10:839. doi: 10.3389/fpls.2019.00839 (PMC6637845; doi:10.3389/fpls.2019.00839)
Supplement: Supplementary file 3 [file Data_Sheet_1.pdf]

Supplementary Material

Integrating Morphological and Physiological Responses of Tomato Plants to Light Quality to the Crop Level by 3D Modelling

J.A. Dieleman*, P.H.B. De Visser, E. Meinen, J.G. Grit, T.A. Dueck

*** Correspondence:** J.Anja Dieleman, anja.dieleman@wur.nl

2 Supplementary Figures

FIGURE S1 | Spectral distribution of the light treatments.

FIGURE S2 | Measured leaf and leaflet angles: LIA= leaf insertion angle; RA= rachis; TLA=top leaflet angle; base= base leaflet angle; middle= middle leaflet angle; outside= outside leaflet angle.
