# Supplementary figures and images for: Integrating Morphological and Physiological Responses of Tomato Plants to Light Quality to the Crop Level by 3D Modeling
Source: Front Plant Sci. 2019 Jul 11;10:839. doi: 10.3389/fpls.2019.00839 (PMC6637845; doi:10.3389/fpls.2019.00839)

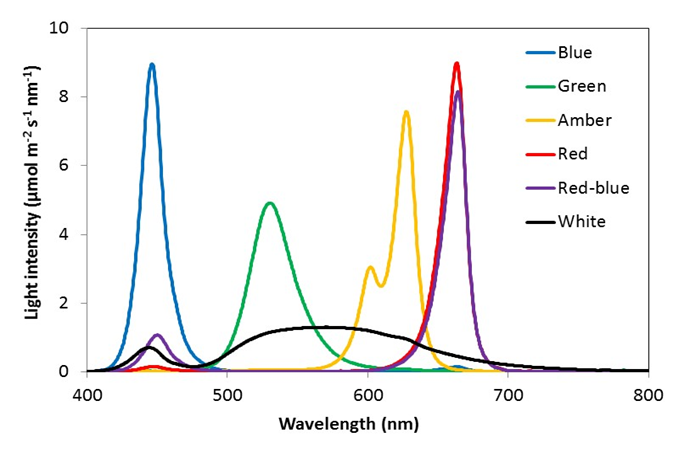

Supplement: Supplementary file 4 [file Image_1.TIF]

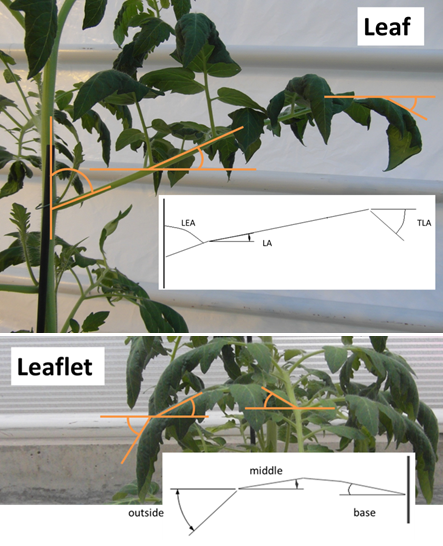

Supplement: Supplementary file 5 [file Image_2.tif]
